# MYCN-amplified spinal ependymoma: a three-patient case series and focussed literature review

**DOI:** 10.1093/jscr/rjag1039

**Published:** 2026-11-14

**Authors:** Finley A J Bettsworth, Helen M Hall, Stewart Griffiths, Mario Ganau

**Affiliations:** Department of Neurosurgery, Oxford University Hospitals NHS Trust, Headley Way, Oxford OX3 9DU, United Kingdom; Department of Neurosurgery, Oxford University Hospitals NHS Trust, Headley Way, Oxford OX3 9DU, United Kingdom; Nuffield Department of Clinical Neurosciences, Level 6, West Wing, John Radcliffe Hospital, Oxford OX3 9DU, United Kingdom; Department of Neurosurgery, Oxford University Hospitals NHS Trust, Headley Way, Oxford OX3 9DU, United Kingdom; Department of Neurosurgery, Oxford University Hospitals NHS Trust, Headley Way, Oxford OX3 9DU, United Kingdom; Nuffield Department of Clinical Neurosciences, Level 6, West Wing, John Radcliffe Hospital, Oxford OX3 9DU, United Kingdom; School of Medicine, BAU International University Batumi, 237 Fridon Khalvashi St, 6010 Batumi, Georgia

**Keywords:** MYCN-amplification, spinal ependymoma, adult spinal cord tumour, molecular classification

## Abstract

MYCN-amplified spinal ependymomas are a rare, recently described aggressive subtype of ependymoma defined by molecular profiling. We present three cases: a 52-year-old man, a 37-year-old woman, and a 28-year-old man presented with progressive neurological symptoms and spinal cord lesions identified on magnetic resonance imaging. These cases demonstrate the challenges in radiological identification and the importance of histological and molecular profiling to guide aggressive treatment of MYCN-amplified spinal ependymomas including surgical resection, radiotherapy, and chemotherapy. Outcomes varied across cases from clinical stability to death, reflecting the heterogeneity of the clinical course. We offer a short literature review to contextualise the cases.

## Introduction

Ependymomas are a histologically and clinically heterogeneous group of rare neuroepithelial tumours arising from ependymal cells throughout the central nervous system (CNS) [1]. These tumours account for 1.4% of all primary CNS tumours, with the majority of adult ependymomas occurring in the spinal cord [2, 3]. Spinal ependymomas are most commonly classical ependymomas and myxopapillary ependymomas; these are indolent Grade II tumours which are often amenable to gross-total resection (GTR) and have a favourable prognosis [1, 4].

A rare and aggressive subtype of spinal ependymoma with anaplastic morphology and a propensity to disseminate has been recently defined through DNA methylation pattern profiling, exhibiting MYCN-amplification [5–7]. The World Health Organization (WHO) Classification of Tumours of the Central Nervous System (2021) integrates anatomical site and histology with molecular data to inform classification and recognizes MYCN-amplified spinal ependymomas as a distinct subtype (SP-EPN-MYCN) [8]. Though there is no specific WHO grade for this tumour type, most exhibit high grade features clinically and on histopathologic analysis, including plentiful mitotic activity, microvascular proliferation, and necrosis [7, 9].

Due to the rarity and recent classification of MYCN-amplified spinal ependymomas as a distinct tumour type, the published literature is limited to individual case reports and small case series. Consequently, its clinical management is poorly characterised, including symptomatic and radiological presentation, optimal surgical strategy, or the efficacy of adjuvant therapies. There is no specific guidance on its management, though prognosis is known to be poor [1]. GTR offers the best prognosis for Grade II and III ependymomas and should be considered early [4, 10].

To address the knowledge gaps in the presentation and management of MYCN-amplified spinal ependymomas, we present a case series of three patients with molecularly confirmed MYCN-amplified spinal ependymomas. We performed a focussed literature review to situate this case series within the existing body of evidence.

## Case series

### Case 1

A 52-year-old man presented with a 3-month history of lower back pain and burning down the anterolateral right thigh. On examination he had tenderness in the L3 area and reduced lumbar flexion due to pain. Magnetic resonance imaging (MRI) of the spine with and without gadolinium contrast revealed two intradural lesions intimately related to the cauda equina nerve roots at the level of the L2 vertebral body and L3/L4 intervertebral disc, measuring 6 and 23 mm, respectively (Fig. 1). These were radiologically in keeping with schwannomas. Multidisciplinary team discussion reached the conclusion that these lesions more likely represent ependymomas. Indeterminate bone lesions within the L1 vertebral body were also noted on MRI and computed tomography (CT) lumbar spine scans which determined to be likely haemangiomas.

**Figure 1:**
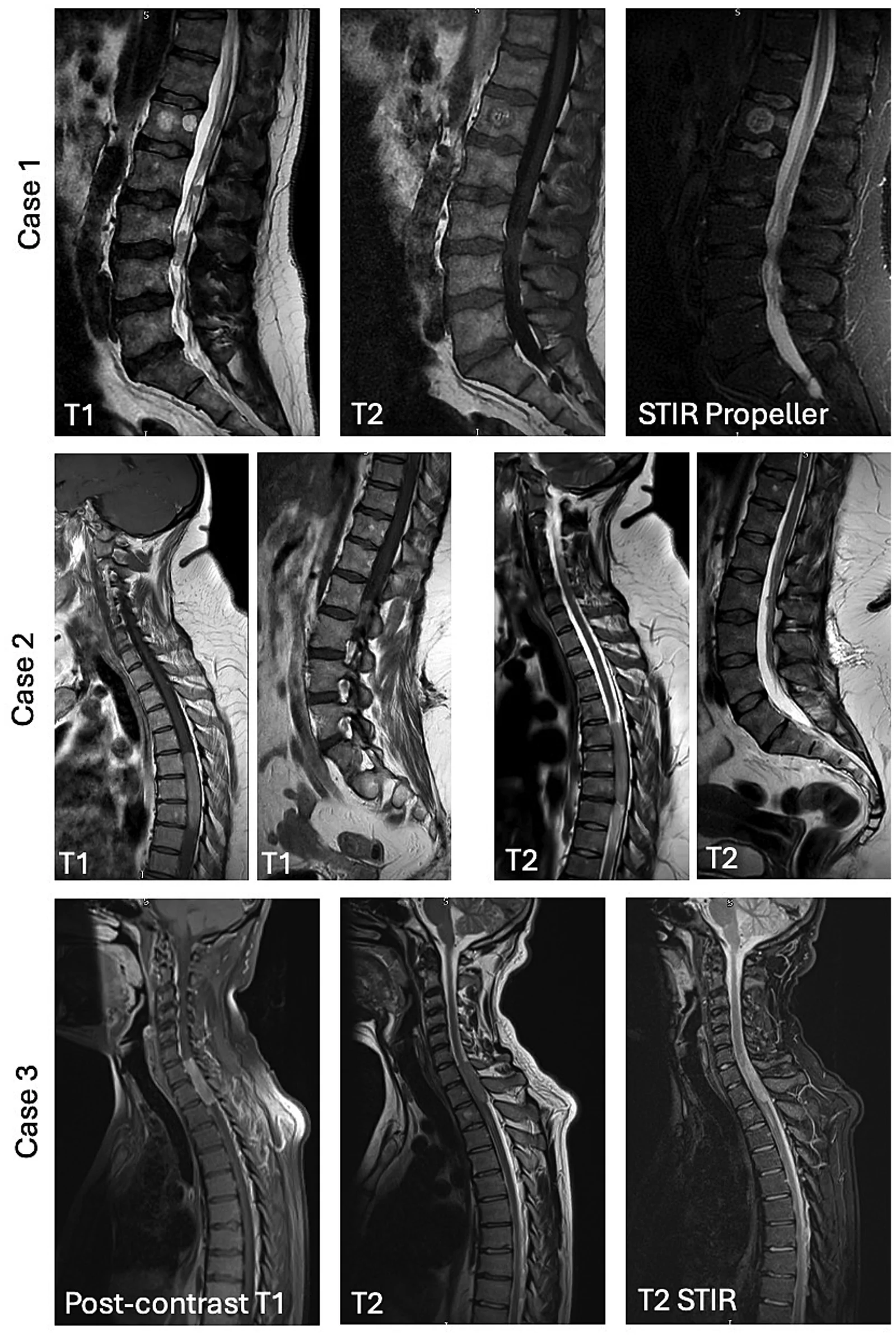
MRI on initial presentation of three cases of MYCN-amplified spinal ependymoma, demonstrating enhancing lesions on T1, T2, and STIR sequences.

The intradural lesions were excised via L2–L4 laminoplasty with no reported post-operative complications. Histology showed a heterogeneous neuroectodermal neoplasm with focally high cellularity, a high MIB1 index, and microvascular proliferation. Morphology and immunophenotyping suggested differentiation as an ependymoma; molecular analysis confirmed a Grade III MYCN-amplified ependymoma.

Small volume residual tumour was noted on MRI 6 weeks post-operatively. Local lumbosacral radiotherapy (54 Gy/30#) was then initiated. Serial MRIs showed a sustained radiological response with reduction of nodule size. However, MRI 1 year after completing radiotherapy showed several tiny foci of enhancement within the thoracic and lumbar spine in keeping with disseminated disease progression.

Following further MDT discussion, he received craniospinal radiotherapy, 36 Gy/20# plus a 14.4 Gy/8# spinal boost, followed by oral etoposide (100 mg once daily). This produced a sustained radiological response after 12 cycles. Seven months later (3 years after diagnosis), MRI demonstrated interval progression, with the largest deposit measuring 1.7 cm. He remains clinically stable with regular surveillance scans (4 years after diagnosis).

### Case 2

A 37-year-old woman presented with 2-month history of sudden left leg numbness and subsequent right leg weakness after a 9-month history of mid-thoracic shooting pains and truncal spasm brought on by lying flat, with post-void hesitancy and dribbling in the weeks preceding admission. Examination revealed bilateral lower limb spasticity with sustained ankle clonus, upgoing plantars, and a sensory deficit with a level of T4. MRI whole spine with and without gadolinium contrast identified a large intradural extramedullary tumour between T5 and T7 with cord compression in keeping with a meningioma, with further intradural nodules at L1 and L2 radiologically characterized as schwannomas/neurofibromas (Fig. 1).

She underwent a T5–T7 laminoplasty and excision of the intradural tumour, achieving GTR of the T5–T7 tumour. She developed a posterior epidural collection post-operatively. Histology identified a moderately cellular and mildly pleomorphic Grade II tumour comprised of spindle cells with psdeudorosettes and no microvascular proliferation, expressing S100 and glial fibrillary acidic protein (GFAP) with focal dot-like EMA positivity. Molecular testing confirmed MYCN-amplification, leading to the diagnosis of MYCN-amplified ependymoma.

Post-operative MRI with contrast and FIESTA sequencing revealed multiple intradural nodules closely relating to the cauda equina roots measuring 2–3 mm, in keeping with drop metastases. Local and national MDT discussion favoured craniospinal irradiation with no further surgery. She completed craniospinal irradiation (35 Gy/21#, thoracolumbar spinal boost 15Gy/9#). MRI 3 months post-radiotherapy (7 months after diagnosis) showed no intracranial disease, no definitive recurrence at primary operative site, and stable lumbar metastatic lesions which have been maintained on serial MRIs until present (2.5 years after presentation).

### Case 3

A 28-year-old man presented following a fall, urinary retention, and lower limb weakness after a 5-week history of back/neck pain and worsening ascending paraesthesia and numbness to a level of T6 on admission. Neurological examination revealed increased tone in all four limbs, bilateral clonus, reduced power in the lower limbs and hyperreflexia. MRI identified a tumour at C7/8 and T1 level, radiologically consistent with a meningioma (Fig. 1). He underwent C6-T1 and upper T2 laminectomy and tumour resection, with near-complete tumour resection and instrumental fusion (C5/C6 lateral mass screws and T1/T2 pedicle screws, rods, and bone graft). Post-operative CT confirmed correct metalwork positioning and MRI showed no residual tumour.

Histology showed a glial neoplasm composed of elongated cells with perivascular pseudorosettes, some microvascular proliferation and necrosis, GFAP expression and dot-like EMA positivity, focal p53 over-expression, and a high MIB1 index, identifying it as a spinal ependymoma. Molecular testing identified MYCN-amplification.

Post-operatively he regained some sensation and movement of the lower limbs, but represented 2 weeks later with wound dehiscence, haematoma, and a deep wound infection (swabs identified *Staphylococcus aureus*) requiring exploration and washout.

He completed cervical spine radiotherapy (50.4 Gy, 28#) 4 months post-operatively. MRI 3 months later showed no local recurrence but multiple new enhancing nodules at T8, T10–L1, and L5 consistent with drop metastases. He represented with urinary retention where an MRI showed confluent deposits causing spinal cord compression. It was decided that he was not a candidate for further radiotherapy or surgery as it would be of little benefit to the patient. Serial MRIs showed progression after 6 months, prompting palliative chemotherapy and he received six cycles of carboplatin and etoposide, and 10 cycles of temozolomide. One year later, MRI showed disseminated disease with cord compression from intradural metastatic lesions at C4, T2/T3, and T4/T5 and increased STIR signal suggesting compressive myelopathy. Further radiotherapy was given for pain control; he declined further treatment and care was transferred to palliative support. He died 8 weeks later (3 years after initial presentation).

## Discussion

MYCN-amplified spinal ependymomas are a recently defined molecular subtype of spinal ependymoma characterised by aggressive clinical behaviour. We describe three patients with molecularly confirmed MYCN-amplified spinal ependymomas who presented with rapid symptom progression, significant neurological morbidity, and early dissemination requiring multimodal treatment.

### Clinical presentation

The three patients in our series presented with symptoms consistent with spinal cord pathology, including back pain, progressive sensory and motor disturbance, upper motor neuron signs, and bladder dysfunction. These findings align with cases in the literature, in which patients commonly present with similar symptoms secondary to spinal cord compression [6, 11–13]. Unlike conventional spinal ependymomas, MYCN-amplified spinal ependymomas demonstrate a marked propensity for early leptomeningeal dissemination [6, 9, 11, 14]. Drop metastases were identified at presentation in Case 2, while the remaining two patients developed disseminated disease during early follow-up.

### Radiological and histological challenges

Radiological diagnosis is challenging in MYCN-amplified spinal ependymomas, with few imaging characteristics to reliably identify this subtype [11]. All three cases were initially considered to represent benign pathologies, with schwannoma and meningioma favoured on initial imaging, and further differentials including cystic dilation of the ventriculus terminalis may be considered based on radiological evidence [15]. This highlights the importance of histopathological and molecular characterization for definitive diagnosis, though radiomics may also represent an emerging mechanism to effectively differentiate ependymomas from other lesions using scans alone [16].

Histological grading has historically been used as the key factor to define management and prognostication of ependymomas. MYCN-amplified ependymomas typically exhibit signs of high-grade ependymal differentiation including high cellularity, perivascular pseudorosettes, positive GFAP, dot-like EMA staining, high MIB1, microvascular proliferation, and necrosis [17]. Recent WHO updates, however, place high emphasis on molecular testing to predict tumour behaviour and inform management [8]. Histology from Case 2 initially indicated a Grade II tumour, but it demonstrated clinical behaviour more aggressive than would be expected with its histological grade; this discrepancy was explained by the identification of MYCN-amplification. This emphasizes the importance of integrated molecular diagnostics, as MYCN-amplification is a more reliable predictor of high-grade ependymoma behaviour than histology alone [6].

### Surgical planning

GTR offers the best prognosis for spinal ependymomas but rarely achieves disease control in MYCN-amplified spinal ependymomas. All three patients underwent tumour resection with GTR achieved in Case 2 and near-complete resection in Case 3, while Case 1 had small-volume residual disease. MYCN-amplified spinal ependymomas grow aggressively and adhere to the spinal cord, complicating resection due to high risks of intraoperative neurological damage. Although resection improved symptoms, further surgical management was precluded by the development of disseminated disease, highlighting the limitations of surgical management in the setting of biologically aggressive spinal ependymomas. In cases of diagnostic uncertainty where radical resection may be unattainable, spinal cord biopsy to ascertain the histological and molecular characteristics of the tumour could be considered to inform management [18].

### Adjuvant therapy and treatment challenges

Although disseminated disease developed in all three cases, disease control has been maintained in Case 2, and Case 1 remains clinically stable despite radiological progression, supporting the role of multimodal therapy in MYCN-amplified spinal ependymoma management. For Grade III or incompletely resected Grade II spinal ependymomas of all subtypes, radiotherapy is recommended with doses of 45–54 Gy [19]. Radical radiotherapy was employed in all cases due to the propensity for leptomeningeal spread. The evidence supporting the use of chemotherapy in spinal ependymomas remains limited [20], and it is typically reserved as a salvage treatment for patients failing surgery and radiotherapy [1]. There is no consensus on therapy choice in MYCN-amplified spinal ependymoma, with a wide range of agents reported in the literature including temozolomide, etoposide, carboplatin, lapatinib, and vorinostat [9, 14]. Poor rates of long-term disease control across the literature demonstrate an unmet need for improved therapeutic strategies. Novel therapies to inhibit MYCN are under investigation, and the role of radiotherapy may expand further with the rise of stereotactic fractionated radiosurgery protocols which take advantage of radio-sensitizers to selectively enhance the treatment effect on tumour cells [1, 21, 22].

### Implications for clinical practice

This case series reinforces the importance of molecular profiling in spinal ependymomas, as the identification of MYCN-amplification heralds an aggressive subtype with prognostic and therapeutic implications. MYCN-amplified ependymomas typically require adjuvant treatments including, but not limited to, craniospinal radiotherapy and chemotherapy. Multidisciplinary team discussion is vital, given the rarity of this subtype and the lack of optimal treatment guidelines.

## Conclusions

We present three cases of MYCN-amplified spinal ependymoma, a rare and highly aggressive ependymoma with a high likelihood of recurrence and leptomeningeal spread. These cases highlight the challenges in diagnosing and managing these tumours with no validated unique imaging findings or consensus on optimal treatment. The routine use of molecular classification is vital for prognostication and treatment planning. However, the heterogeneity in clinical course of our patients illustrates the challenge in predicting tumour behaviour, demonstrating the need for larger studies to identify factors driving variability in outcomes and to define guidelines for clinical practice.
